# Trends and Economic Impact of Hip and Knee Arthroplasty in Central Europe: Findings from the Austrian National Database

**DOI:** 10.1038/s41598-018-23266-w

**Published:** 2018-03-16

**Authors:** Lukas Leitner, Silvia Türk, Martin Heidinger, Bernd Stöckl, Florian Posch, Werner Maurer-Ertl, Andreas Leithner, Patrick Sadoghi

**Affiliations:** 1 Department of Orthopedics and Trauma, Medical University of Graz, Graz, Austria; 2 Federal Ministry for Health and Women’s Affairs, Vienna, Austria; 3 Department of Orthopedic Surgery, LKH Klagenfurt, Klagenfurt, Austria; 4 Division of Oncology, Department of Internal Medicine, Medical University of Graz, Graz, Austria

## Abstract

Arthroplasty registers were originally established in Scandinavia to receive clinically relevant information from pooled data, to improve quality and reduce revision surgeries, with socioeconomic benefit. In Austria, where the highest rate of total knee arthroplasties (TKA) per inhabitant of all OECD countries was reached in 2014, arthroplasties are centrally reported since 2009. Study purpose was to perform the first analysis of the Austrian database, aiming to obtain data on trends in arthroplasty in Austria over time in relation to demographic development. Between 2009 and 2015 an almost continuous increase of total hip arthroplasties (THA; 18.052) by 14% and TKA (17.324) by 13% were observed, representing 210 THA and 202 TKA per 100k inhabitants in 2015. A similar increase was found for revision surgeries, with 1.290 re-implanted THA (7.1% of all THA) and 919 re-implanted TKA (5.3% of all TKA) in 2015. Implantation of mega or tumor prosthesis for the knee and hip joint remained constant and was mainly performed in two university hospitals. Patellar resurfacing decreased by 31.6%. Demographic development will further increase the number of primary and revision surgeries. Inclusion of more detailed information on used and revised components was established and will improve efficacy in quality control.

## Introduction

The first National Arthroplasty Register was introduced in Sweden in 1975, followed by many other countries. Arthroplasty registration can lead to identification of poorly-performing implants and surgical procedures after market introduction, so that healthcare providers may improve surgical practice by choosing better performing products^1^. In Sweden, a significant reduction of revision surgeries is considered to be a result of the register^2^. Effective register recording requires a complete, yet practicable information-set, as well as a high level of input compliance, which in turn demands the management of complex political, economic, and practical challenges^3^.

Austria is a Central European Country with a nationally funded health system. Under the aspect that the highest rate of total knee arthroplasties (TKA) per inhabitant of all OECD countries in 2014 was reached in Austria^4^, knowledge on numbers of surgeries in this field seems to be important for quality control and anticipation of future trends. The total number of total hip arthroplasty (THA) and total knee arthroplasty (TKA) and implant related revision surgeries in this field is recorded in a standardized manner by the national health authorities since 2009. The recorded data include age group, sex, and in which of the nine Austrian federal provinces it was included. The implementation of the registry in Austria is not a classical arthroplasty register as compared to Sweden or Germany but an analysis of the performance-based hospital financing system.

The purpose of this study was to provide the first analysis of the Austrian database, representing a highly developed industrialized country, with the focus to present systematic data on trends in arthroplasty of the hip and knee from 2009 to 2015 in relation to the demographic development.

## Methods

Number of joint replacements and revision surgeries of the hip (primary arthroplasty, tumor/resection replacement, explantation, cement spacer implantation/explantation, re-implantation, exchange of not bone-anchored implant components) and knee joint (primary arthroplasty, tumor/resection replacement, explantation, cement spacer implantation/explantation, re-implantation, exchange of not bone-anchored implant components, patellar surface replacement) were centrally registered by the Austrian health authorities with respect to the performance-based hospital financing system^5^. The number is based on service codes transmitted by healthcare providers to receive payment for the provided medical services. Total number, sex, age group in 5 years steps, and the province were recorded. Descriptive analysis of the database as well as subgroup analysis concerning patients´ age, sex, and chronological sequence was performed. Extraction of implantation data for singular hospitals was not possible from the data. Comparison to data on demographic trends in Austria, which are published yearly by “Statistik Austria” (formerly, Austrian Statistical Office) was performed^6^.

### Statistical methods

Descriptive statistics were calculated from data delivered by the national health authorities and “Statistik Austria” institution. The calculated mean age was estimated assigning the median age for the age group to all patients belonging to an age group. IBM SPSS Statistics 20 was used for data analysis.

### Ethics statement

No Institutional Review Board approval was required for this project.

## Results

### Analysis of primary arthroplasties

**Figure 1:**
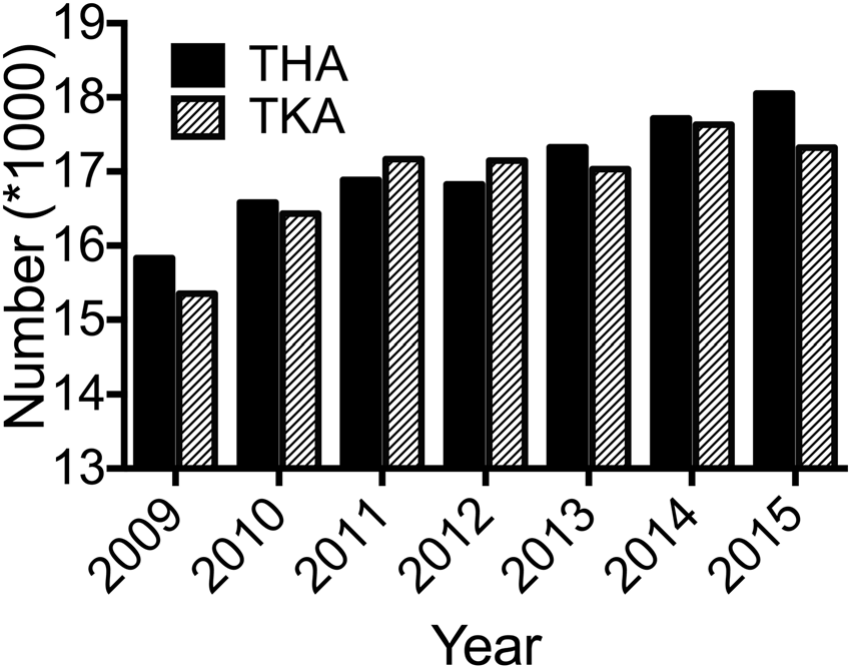
Primary THA and TKA per year in Austria.

Between 2009 and 2015 an almost continuous increase in the number of hip and knee arthroplasties was found. In detail, the number of primary THA increased by 14% (n = 15,834 in 2009 and n = 18,052 in 2015) and the number of primary TKA increased by 13% (n = 15,350 in 2009 and n = 17,324) over this period, respectively (Fig. 1, Table 1). This represents 210 primary THA and 202 primary TKA per 100.000 inhabitants in 2015. Ninety-two percent of THA and 97% of TKA were performed in the age group 50-90 between 2009 and 2015 (Suppl. Figure 1). Between 2009 and 2015 the population aged 50-90 increased only by 1.1% to 3.3 million people, the population older than 65 years in Austria increased by 9.3%, and the population older than 70 years increased the strongest by 19.8%^6^. The mean age of patients receiving THA (67.1 to 67.4 years) and TKA (69.7 to 69.5 years) remained relatively constant between 2009 and 2015 (Table 1), whereas the corresponding mean age in the Austrian general population increased during this period (41.2 to 42.3 years)^6^.

**Table 1:** Absolute numbers of primary implantation and re-implantation of THA and TKA, including sex and age, from 2009 to 2015 in Austria.

Year		2009	2010	2011	2012	2013	2014	2015
Primary THA	Male	6760	7106	7330	7380	7404	7743	7831
	Female	9074	9479	9560	9446	9921	9974	10221
	Total	**15834**	**16585**	**16890**	**16826**	**17325**	**17717**	**18052**
	*Age (y; mean)*	*67,1*	*66,9*	*66,9*	*67,1*	*67,3*	*67,2*	*67,4*
Primary TKA	Male	5625	6064	6467	6571	6353	6597	6703
	Female	9725	10367	10702	10579	10677	11034	10621
	Total	**15350**	**16431**	**17169**	**17149**	**17030**	**17631**	**17324**
	*Age (y; mean)*	*69,7*	*69,7*	*69,7*	*69,4*	*69,5*	*69,4*	*69,5*
Reimpl. THA	Male	419	389	429	418	371	437	543
	Female	539	532	541	525	498	484	747
	Total	**958**	**921**	**970**	**943**	**869**	**921**	**1290**
	*Age (y; mean)*	*70,0*	*69,7*	*69,9*	*70,0*	*70,6*	*71,1*	*70,8*
Reimpl. TKA	Male	266	275	289	310	335	341	347
	Female	485	506	510	493	550	540	572
	Total	**751**	**781**	**799**	**803**	**885**	**881**	**919**
	*Age (y; mean)*	*70,4*	*69,6*	*69,8*	*69,8*	*69,9*	*69,6*	*70,2*

### Analysis of revision arthroplasties

**Figure 2:**
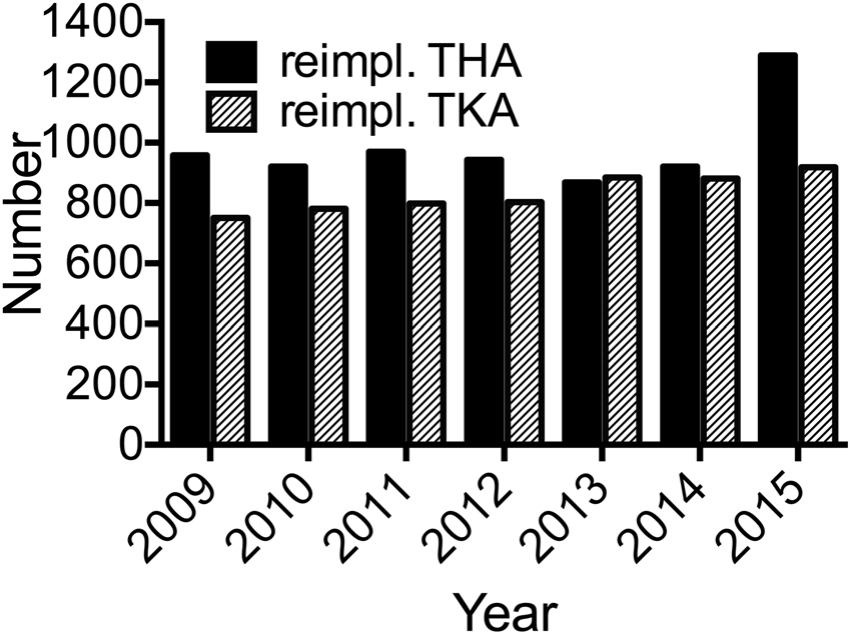
Re-implantation THA and TKA per year in Austria.

An increase over time was also found for revision surgeries, as 1290 THA re-implantations (7.1% of primary hip arthroplasties, increase of 34.7% between 2009 and 2015) and 919 TKA re-implantations (5.3% of primary knee arthroplasties, increase of 22.4% between 2009 and 2015) were performed in 2015 (Fig. 2, Table 1). In 2015 the calculated mean age of patients receiving re-implantation surgery in Austria was 70.8 years for THA and 70.2 years for TKA (Table 1; Suppl. Figure 2).

### Relation to demographic development

**Figure 3:**
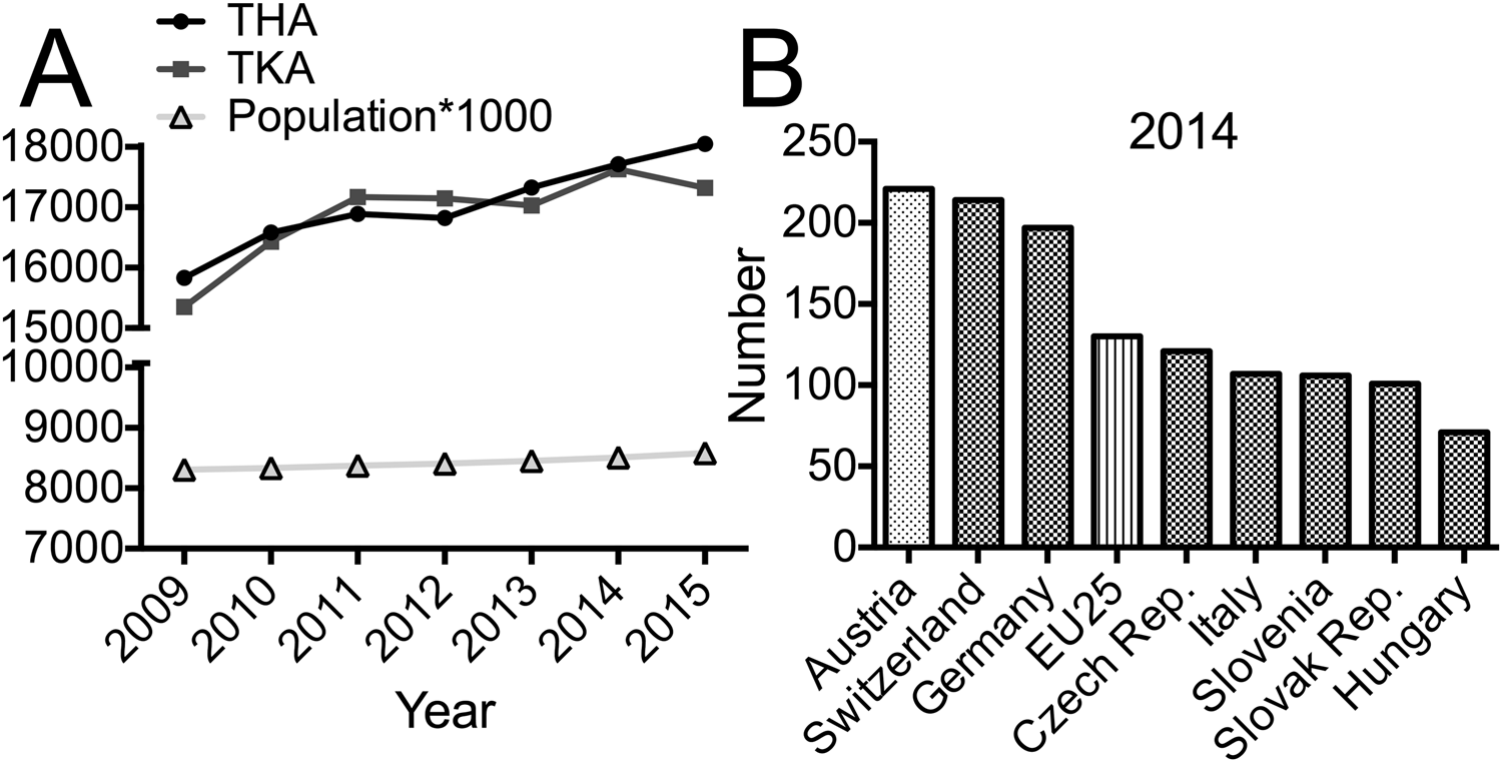
(**A**) Development of implanted primary THA and TKA and Austria’s population (adapted from^6^) during the study observation time. (**B**) Knee replacement surgery 2014, Austria and neighboring countries; adapted from^4^.

The number of Austria’s inhabitants increased only moderately during the period of investigation (3%; n = 8.335.003 in 2009 and n = 8.584.926 in 2015)^6^ compared to the increase of primary THA (14%) and TKA (13%) (Fig. 3A). The peak of this development resulted in the highest rate of TKA per inhabitant of all OECD countries in 2014 (Fig. 3B)^4^.

### Subanalysis of miscellaneous arthroplasties

Exchange of none-bone-anchored implant components performed after THA (i.e. inlay, head) remained on a steady state during the observation time (n = 681 in 2015; increase of 5.6%). Registration of spacer implantation of the hip was first initiated in 2015 where a total number of 114 spacers were implanted, most often in the age group between 70 and 74 years. During the period of investigation, the number of implanted mega prostheses or tumor prosthesis for knee (n = 112 in 2015) and hip (n = 184 in 2015) remained stable (Suppl. Figure 3A). Implantation was mainly performed in two regions with orthopedic tumor centers in Austria (i.e. Vienna, Styria; Suppl. Figure 3B). Two implantation peaks were found for mega prosthesis and tumor prosthesis for knee and hip, the first around 15–20 years and the second in the population 60 years and older (Suppl. Figure 3C). The only implant type that clearly decreased in case numbers during the observation period was patellar resurfacing with a decrease of 31.6% between 2009 (n = 1983) and 2015 (n = 1356) (Suppl. Fig. 4).

## Discussion

This manuscript provides the first analysis of a detailed report on the Austrian National database on knee and hip arthroplasty, in comparison with demographic development in Austria from 2009 to 2015.

We explored an increase in almost all implant-related surgeries of the knee and hip joint in Austria during the observation time from 2009 to 2015. Corresponding increases can be found in similar studies from most developed countries (Fig. 1)^7,8^. The increased number of implanted THA by 14% and the increased number of TKA by 13% over this period represents 210 THA and 202 TKA in 100k inhabitants in 2015. This number is relatively high in comparison to numbers from other countries previously published by our study group^9^. Demographic change, especially increasing proportion of elderly people, might be a main reason for this increase in operations. Studies in Germany, a neighboring country to Austria with a similar demographic change, have shown that the increase in age leads to an increase in multimorbidity and especially to an increase in the prevalence of osteoporosis which triggers musculoskeletal diseases^10–12^. On the other hand, it has been shown that technical innovation led to reduced surgical risk, resulting in lower threshold for indicating arthroplasties, and thus enabling an increased number of surgeries especially in elderly patients^13^. Our data revealed that the increment of implanted THA and TKA is disproportional compared to increase in Austria’s population during the observation time (Fig. 3A). The OECD recently published, that Austria had the highest rate of TKA per inhabitant of all OECD countries in 2014^4^ (Fig. 3B).

As stated before, demographic change alone can only represent part of explanation for increasing number of implanted prosthesis^4^. In part, ongoing development of medical care also seems to be responsible for increased intervention numbers. On the other hand, environmental development may lead to economic growth pressure, resulting in higher implant numbers than explored for comparable neighboring countries (Fig. 3B). Such a development should be avoided in a health system since it may result in ‘overtreatment’ with arthroplasties.

Mean age in Austria’s population increased, whilst mean age of patients undergoing primary THA and TKA remained on steady state during the observation period, suggesting a slight trend towards younger arthroplasty receivers. A similar phenomenon was explored in the United States and Canada in earlier studies^8,14^.

Especially the disproportionately high increase in revision surgeries after THA and TKA (Fig. 2) seems to be a result of the combination of trends addressed above, higher implantation numbers, younger receivers, and increasing life expectancy, altogether resulting in an increased population at risk. Earlier authors have raised concern on survivorship of primary TKA and revision surgeries in younger patients, for which higher risk of early periprosthetic joint infection and aseptic mechanical failure after primary implantation as well as higher rates of aseptic failure after revision have been described^15,16^.

Implantations of mega prosthesis or tumor prosthesis for the knee and hip are usually performed in tertiary centers, where the underlying disease can be treated^17^, resulting in concentration of implant numbers in Vienna and Styria, where skeletal oncology centers are located (Suppl. Figure 3B). This data interpretation is supported by the two age peaks for both, implanted mega prosthesis or tumor prosthesis for the knee and hip, congruent to age peaks for incidence of osteosarcoma and Ewing sarcoma in young patients and chondrosarcomas and bone metastases in the elderly (Suppl. Figure 3C)^18–20^.

The Austrian database reveals a continuous decrease of 31.6% in the number of patellar resurfacing performed during the observation time. A similar trend has been described by our group in a meta-analysis of arthroplasty registers, as, for example in Sweden, whereas TKA is nowadays mostly performed without patella buttons^9^. The benefit of patellar resurfacing is discussed controversially in the scientific society; whilst the majority of surgeons (>90%) routinely resurface the patella in North America, most Austrian surgeons seem to only selectively resurface based upon patient factors or during revision surgeries^21^.

### Study Limitations

The Austrian arthroplasty registry is based on data which are transmitted mainly for deposit with the public health system. Although this implicates high completeness of our dataset, it has lower granularity than conventional prosthesis registers on the patient-level as provided by Sweden or Germany. Although, networking back on individual cases (for inspection purposes) is possible, the data do not contain additional information except for the parameters presented. Therefore, no information is available on patients clinical condition, how surgery was performed, implant type and manufacturer, patient reported outcome scores, or follow up, (etc.) presenting a major limitation for more detailed investigations. However, it is possible to screen for higher revision or complication rates in order to perform an on-site peer review audit organized by the Austrian ministry of health.

In 2015, the service codes for revised knee or hip arthroplasty were further specified on the component of the prosthesis, which was revised. For comparability with data from the years before, the numbers for new codes were summed up. Notably there was a peak in number of THA re-implantations in 2015 (Fig. 2A), which could result from transmission of service codes for each component when the complete system was changed. This would represent a reporting bias. This effect was not found for TKA re-implantations in 2015.

The authors believe that the implantation number for mega prosthesis reported to the Austrian health authorities is too high. This could represent a reporting bias, resulting from transmission of service codes for mega prosthesis when large revision surgeries of TKA or THA were performed.

## Conclusion

Many parallels to earlier published databases and register results can be drawn from this Austrian National database analysis, and underline the validity of our data. A common consensus could be that numbers of arthroplasties are still increasing in developed countries; the peak in implantation numbers for primary implantations and revision surgeries after arthroplasty seems not to be reached yet. Knowledge on numbers of surgeries in this field is important for quality control in the Austrian public health system. Inclusion of more detailed information on used and revised components, as it is established since 2015, will improve efficacy in quality control.

## Electronic supplementary material

Supplementary Information
