# Supplementary material for: Trends and Economic Impact of Hip and Knee Arthroplasty in Central Europe: Findings from the Austrian National Database
Source: Sci Rep. 2018 Mar 16;8:4707. doi: 10.1038/s41598-018-23266-w (PMC5856851; doi:10.1038/s41598-018-23266-w)

**SUPPLEMENTARY INFORMATION**

**Trends and Economic Impact of Hip and Knee Arthroplasty in Central Europe: Findings from the Austrian National Database.**

Lukas Leitner^1^, MD, PhD, Silvia Türk^2^, MD, Martin Heidinger^2^, Bernd Stöckl^3^, MD, Florian Posch^4^, MD, MSc, Werner Maurer-Ertl^1^, MD, Andreas Leithner^1^, MD, Patrick Sadoghi^1^, MD, PhD*

^1^ Department of Orthopedics and Trauma, Medical University of Graz, Graz, Austria

^2^ Federal Ministry for Health and Women's Affairs, Vienna, Austria

^3^ Department of Orthopedic Surgery, LKH Klagenfurt, Klagenfurt, Austria

^4^ Division of Oncology, Department of Internal Medicine, Medical University of Graz, Graz, Austria

**SUPPLEMENTARY FIGURE LEGENDS**

**Suppl. Figure 1.** All primary THA and TKA from 2009 to 2015 in Austria grouped by age.

**Suppl. Figure 2.** All re-implantation THA and TKA from 2009 to 2015 in Austria grouped by age.

**Suppl. Figure 3A.** Mega prosthesis THA and TKA per year in Austria. **B.** All tumor-/resection-THA from 2009 to 2015 in Austria grouped by region. **C.** Tumor-/Resection-THA and TKA from 2009 to 2015 in Austria grouped by age.

**Suppl. Figure 4.** Patellar resurfacing per year in Austria.

**Suppl. Figure 1.**





**Suppl. Figure 2.**


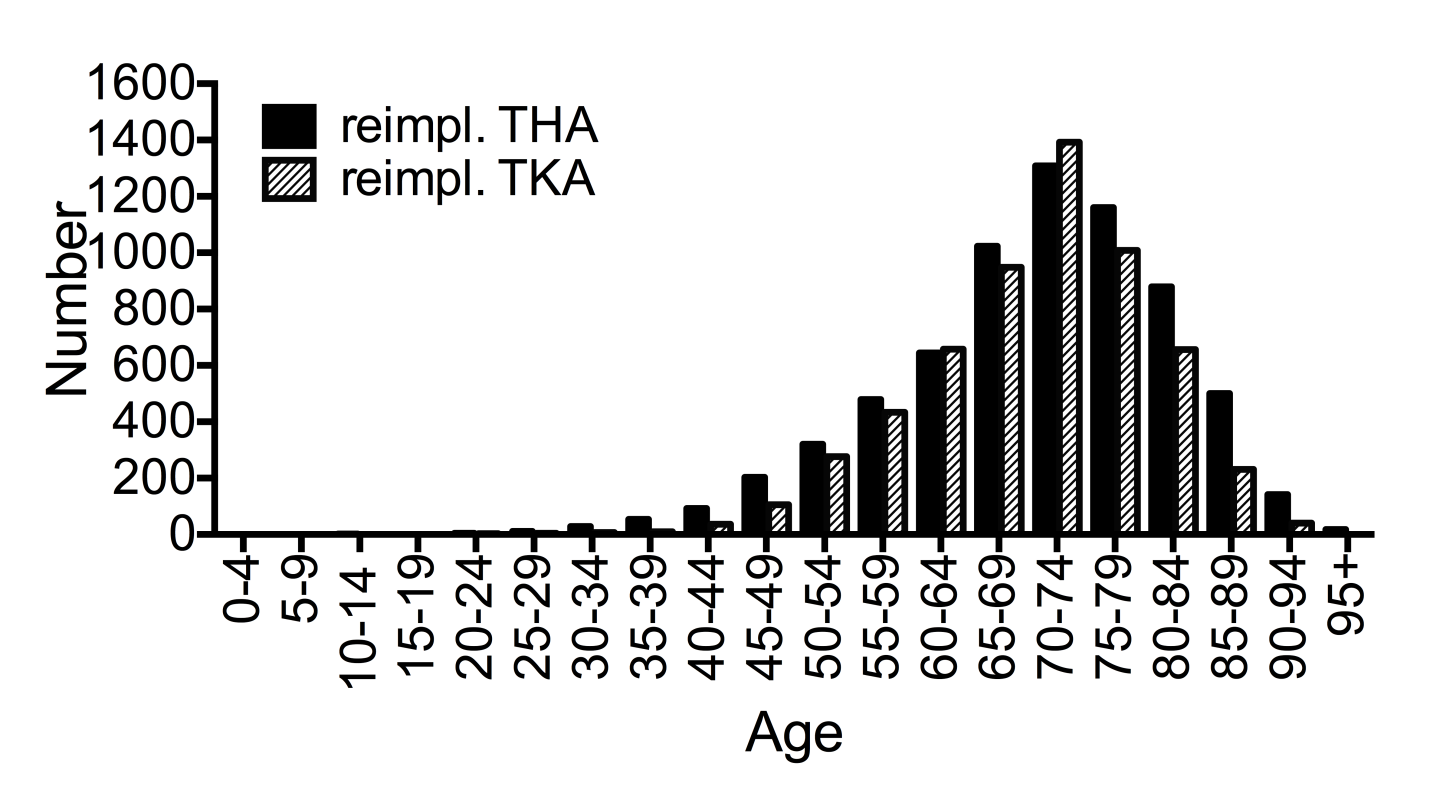


**Suppl. Figure 3.**


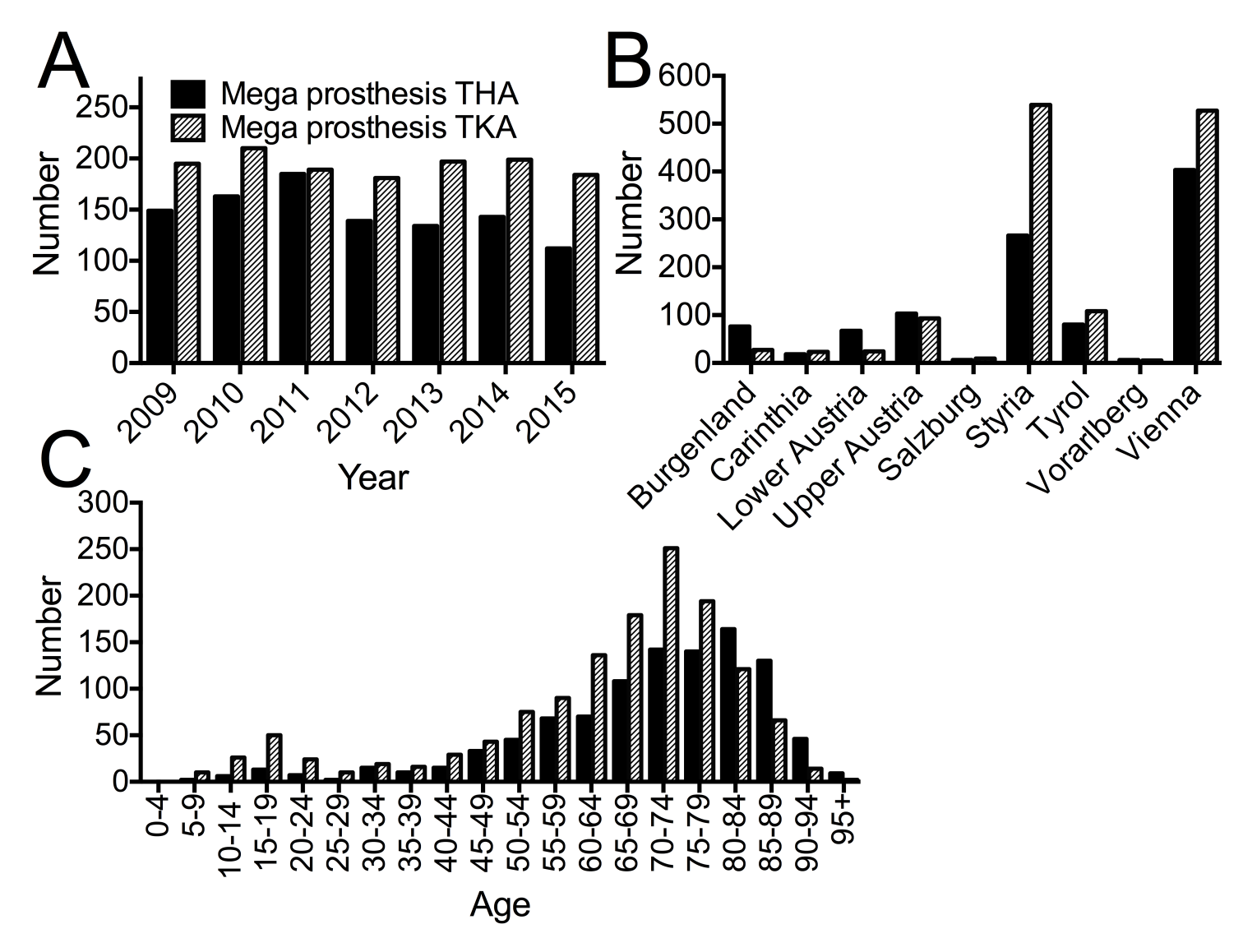


**Suppl. Figure 4.**


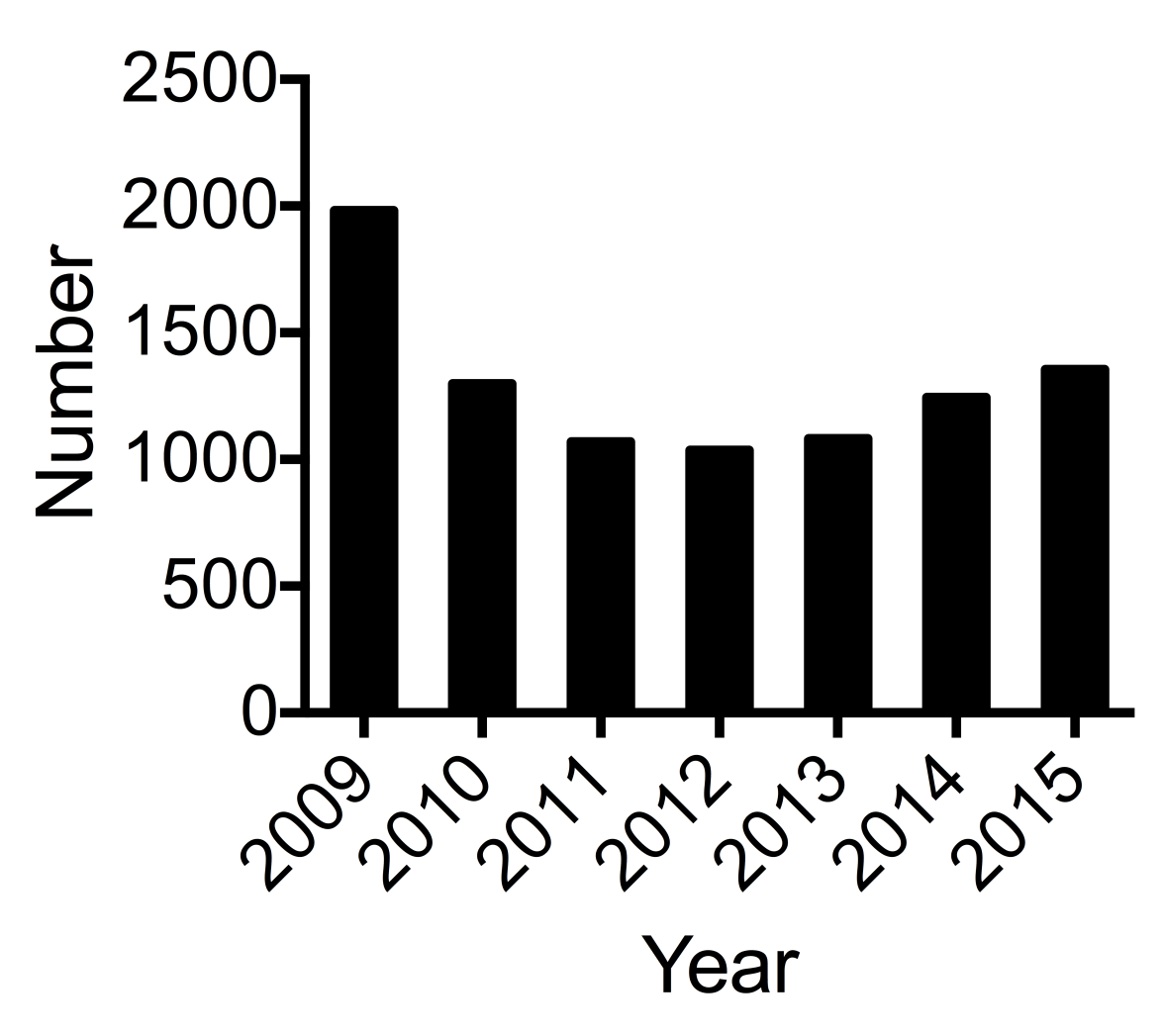

Supplement: Supplementary file 1 — Supplementary Information [file 41598_2018_23266_MOESM1_ESM.docx]
